# Nutritional Interventions in Head and Neck Cancer Patients Undergoing Chemoradiotherapy: A Systematic Review and Meta-Analysis

**DOI:** 10.3390/healthcare13243324

**Published:** 2025-12-18

**Authors:** Sneha Patnaik, Jiun-Yi Wang, Fawziyyah Usman Sadiq, Khemraj Sharma

**Affiliations:** 1 Department of Healthcare Administration, Asia University, Taichung 413305, Taiwan; 2 Department of Medical Research, China Medical University Hospital, China Medical University, Taichung 404333, Taiwan; 3 International Relations Office, Kalinga Institute of Industrial Technology-DU, Bhubaneswar 751024, India

**Keywords:** head and neck cancer, nutritional intervention, chemoradiotherapy

## Abstract

**Background:** Head and neck cancer patients frequently encounter nutritional deterioration, culminating in poor clinical and treatment-related outcomes and reduced quality of life. This systematic review and meta-analysis aim to examine the effects of non-invasive nutritional interventions on nutritional status and clinical, biochemical, and patient-reported outcomes. **Methods:** A comprehensive literature search across five databases (PubMed, CINAHL, ProQuest, Medline, and Scopus) was carried out to identify potentially relevant randomized control trials published in English between 2019 and 2024. Screening, extraction of data, and quality check were carried out separately by two reviewers. The Joanna Briggs Critical Appraisal tools assessed the quality of the included studies and evidence certainty was appraised using the GRADE framework. Depending on the amount of heterogeneity present, a random or fixed-effects model was used to conduct the meta-analysis. **Results:** Eleven studies were included, involving 1000 participants. Pooled estimates showed significant effects on weight (SMD = 0.171, 95%CI: 0.008, 0.335, *p* = 0.04), serum albumin (SMD = 0.539, 95%CI: 0.150, 0.927, *p*= 0.007), and patient-generated subjective global assessment score (SMD = −0.518, 95%CI: −0.931, −0.106, *p* = 0.014) in the intervention group compared to controls. Bias concerns were observed in some studies, largely stemming from inadequate blinding and deviations from intention-to-treat analysis. Evidence certainty ranged from moderate to very low. **Conclusions:** Non-invasive, patient-directed nutritional interventions may lead to clinically meaningful benefits in patients with head and neck cancer receiving chemoradiotherapy, particularly for the maintenance of body weight and nutritional status. However, robust, adequately powered trials with standardized reporting of intervention components and outcome measures are needed in the future to strengthen the evidence base for clinical application.

## 1. Introduction

Carcinomas of the head and neck are ranked seventh amongst all cancer types worldwide, with an incidence of 947,211 cases annually and approximately 482,428 deaths, accounting for 4.9% of all cancer-related mortalities in 2022 [1]. Recent global estimates also report that the age-standardized prevalence rate of head and neck cancer (HNC) was 87.2 per 100,000 population in 2021 among individuals aged 40–64 years [2]. Due to the tumor’s anatomical location compromising mastication, swallowing, and oral intake, along with systemic metabolic alterations, intensive treatments, and their associated side effects, those affected with malignancies of the head and neck often encounter challenges in maintaining adequate nutrition. Therefore, they are among the most nutritionally vulnerable cancer populations [3,4,5,6]. Nearly 40% of patients are malnourished even before initiating treatment and tend to experience progressive nutritional decline throughout their treatment trajectory and follow-up [7,8].

Malnutrition in HNC patients is exacerbated by an increased prevalence of nutrition impact symptoms such as mucositis, xerostomia, nausea, and loss of appetite, commonly induced by chemoradiotherapy (CRT). These symptoms further impede oral intake, leading to unintentional loss of weight and muscle depletion. Such nutritional deterioration is strongly linked to poor clinical outcomes, reduced physical functionality, lower treatment tolerance, and increased morbidity and mortality [9,10]. Since malnutrition significantly contributes to poor prognosis and high disease burden in HNC patients, providing effective nutritional interventions is crucial to improving treatment outcomes and enhancing quality of life (QoL). Moreover, approximately 10–20% of HNC-related deaths are attributed to malnutrition rather than the cancer itself, emphasizing the importance of addressing nutritional deficits [11].

Nutritional interventions for HNC patients encompass a range of strategies, broadly classified into invasive and non-invasive approaches. Non-invasive methods include nutritional counseling (NC), dietary modification, oral nutritional supplements (ONS), and immunonutrition [12]. NC, regarded as the cornerstone of nutritional support, provides patients with tailored dietary advice alongside ongoing nutritional assessments carried out at different stages of treatment. ONS consist of nutrient-dense formulations designed to improve overall intake and prevent further weight loss. Immunonutrition uses specialized nutrients, like omega-3 fatty acids, arginine, glutamine, and nucleotides, to regulate immune function, reduce inflammation, and mitigate treatment-related toxicities. These are patient-directed strategies that can be feasibly implemented at home or outpatient settings, promoting patient autonomy [13,14]. Invasive approaches like enteral and parenteral nutrition are implemented under strict medical supervision in more severe cases characterized by significant impairment in oral intake. These methods are mostly reserved for patients with severe dysphagia or profound weight loss, wherein, non-invasive techniques prove inadequate [15]. Although these interventions are vital for preventing further decline, preference for non-invasive, patient-centered approaches remains strong due to their feasibility and potential for enhancing patient adherence and comfort.

Although some randomized controlled trails (RCTs) have demonstrated improvements in body weight, caloric intake, and treatment tolerance [16,17], others reported negligible or inconsistent effects across nutritional and clinical outcomes [18]. This variability reflects differences in intervention components, modes of delivery, and outcome assessment methods, highlighting the need for a rigorous evidence synthesis.

To date, existing reviews have been limited in scope or methodological rigor. Previous systematic reviews [19,20] have examined nutritional interventions in HNC but none have provided a comprehensive meta-analysis exclusively targeting non-invasive, patient-directed interventions among those undergoing chemoradiotherapy (CRT). Existing reviews were largely narrative [21,22] or included mixed heterogeneous treatment modalities, and no prior synthesis has offered pooled estimates derived solely from randomized controlled trials. Furthermore, evidence gaps remain in understanding the effectiveness of these interventions on clinical, biochemical, and patient-reported outcomes, and earlier work did not incorporate systematic evaluations of the risk of bias or certainty of evidence. To address these gaps, the present review provides an updated and methodologically rigorous synthesis, including meta-analyses and GRADE-based certainty assessments, focusing specifically on non-invasive nutritional interventions in HNC patients receiving CRT.

## 2. Methods

This systematic review and meta-analysis used the Preferred Reporting Items for Systematic Reviews and Meta-Analyses (PRISMA) guidelines, 2020 iteration [23]. The protocol for this review was prospectively registered in the PROSPERO International Prospective Register of Systematic Reviews (registration number: CRD42024610805) to ensure methodological transparency and adherence to best reporting practices.

### 2.1. Search Strategy and Study Selection

A thorough literature search was carried out across five electronic databases: PubMed, Medline, Cumulative Index of Nursing and Allied Health Literature (CINAHL), Scopus, and ProQuest. Citation searching was performed by reviewing the reference lists of eligible articles and relevant reviews. This led to the identification of two additional studies that could not be retrieved during the initial database search due to variations in indexing and keywords. The search included a combination of several keywords: (“head and neck cancer” OR “head and neck carcinoma” OR “head and neck neoplasm” OR “head and neck malignancy” OR “head and neck tumor”) AND (“nutritional intervention” OR “nutritional support” OR “dietary therapy” OR “dietary counseling” OR “nutritional counseling” OR “oral nutritional supplement” OR “nutritional advice” OR “immunonutrition” OR “immune enhancing nutrition”) AND (“nutritional status” OR “weight loss” OR “body weight” OR “nutritional intake” OR “biochemical marker” OR “inflammatory marker” OR “hemoglobin” OR “albumin” OR “pre-albumin” OR “quality of life”) AND (“randomized controlled trial” OR “controlled clinical trials” OR “random allocation”). The full search strategy is provided in the Supplementary Material (Table S1).

The literature search was restricted to studies published between 2019 and September 2024. This timeframe was selected to ensure that the synthesized evidence reflects contemporary nutritional care standards in cancer patients. Nutritional interventions delivered during chemoradiotherapy closely follow the clinical practice guidelines available at the time, and the design and conduct of interventional studies typically follow these prevailing recommendations. The publication of the comprehensive ESPEN guideline on nutrition in cancer in 2017 [24] marked an important shift in international practice, and subsequent clinical trials were influenced by its recommendations. Considering the lag required for guideline adoption, study execution, and manuscript publication, establishing the search window beginning approximately one to two years after this major guideline release is methodologically reasonable.

### 2.2. Inclusion and Exclusion Criteria

Randomized control trials published in English language in peer-review journals were deemed fit for inclusion if they met the following criteria: (1) focused on HNC patients undergoing CRT alone or in combination with other treatment modalities, (2) investigated non-invasive, patient-directed nutritional interventions such as oral nutritional supplements, nutritional counseling, and immunonutrition, (3) addressed at least one of the following outcomes: nutritional outcomes, biochemical markers, PG-SGA score, clinical and patient-reported outcomes, and psychological outcomes, (4) were written in English, (5) were published in peer-reviewed journals, and (6) if they had full-text eligibility. Studies were excluded if they (1) focused on malignancies other than HNC, (2) participants underwent surgical treatment instead of CRT, (3) did not address nutritional or clinical outcomes, (4) reported invasive nutritional interventions (enteral or parenteral), or if they (5) were editorial, commentaries, case studies, research protocols, or systematic reviews.

### 2.3. Data Screening and Extraction

**Figure 1:**
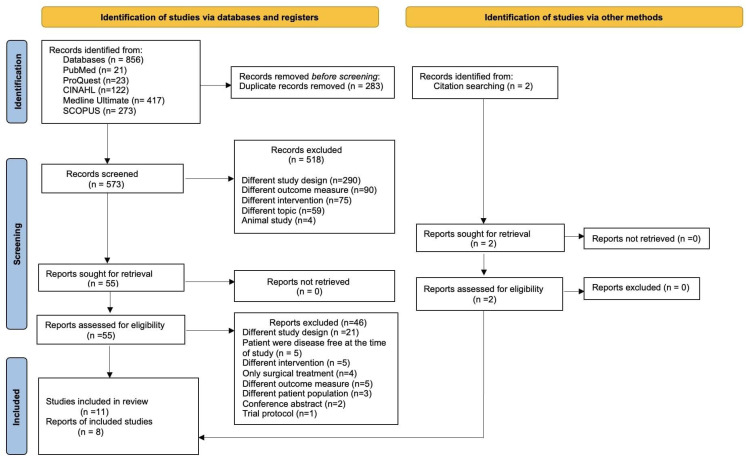
PRISMA flow diagram for the selection of studies.

Prior to screening and data extraction, duplicate references were systematically identified and removed using Endnote 20. Figure 1 illustrates the article selection process. Two reviewers (S.P. and F.S.) individually reviewed the title and abstracts of each identified paper to ascertain eligibility for full-text screening. Full texts of each eligible article were retrieved for further review. Each article was then screened independently by the reviewers and assessed in its full-text form to determine the final pool of studies to be included in the review through discussions and consensus. Disagreements were resolved collaboratively, and a third reviewer (K.S.) was called upon when consensus could not be reached. Both reviewers extracted data on the author, year, country, sample size, treatment received, intervention type, tools for the measurement of the outcome and main findings from each study and recorded the information using a Microsoft Excel 2010 spreadsheet (Microsoft Corp., Redmond, WA, USA) to facilitate organization and analysis. The reviewers met regularly during the data extraction process to discuss and resolve any discrepancies.

### 2.4. Quality Appraisal

The Joanna Briggs Institute (JBI) checklist for critical appraisal was used to assess the risk of bias of the selected studies [25]. The checklist included thirteen questions evaluating key domains such as randomization, allocation concealment, blinding, completeness of follow-up, presence of intention-to-treat analysis, reliability of methods used to measure outcomes, and appropriateness of the statistical analysis and trial design used. For each item, the response was coined as “yes,” “no,” or “unclear”. Two independent reviewers carried out the quality assessment, addressing any disagreements through repeated discussions. To visually summarize the risk of bias across the studies, a traffic light plot, according to the responses from the JBI checklist, was generated using the Robvis tool [26]. The tool was custom-mapped to reflect the individual JBI checklist items for each domain. Certainty of evidence was assessed using the Grading of Recommendations Assessment, Development, and Evaluation (GRADE) [27]. The certainty of each outcome was downgraded if the included studies were identified to have a high risk of bias.

### 2.5. Statistical Analysis

Comprehensive Meta-Analysis (CMA) software (version 3.0, Biostat, Englewood, NJ, USA) was used to perform meta-analytic computations. To ensure inter-rater reliability in the study selection and quality assessment process, the kappa statistic was used. Using the Inverse Variance method, the standardized mean differences (SMDs) and 95% confidence intervals (CIs) were computed for continuous outcomes. Pre- and post-treatment means, standard deviations (SDs), and sample sizes for each parameter were entered into the software. For paired data, the correlation coefficient between pre- and post-treatment measures was assumed to be 0.5 unless otherwise specified in the studies.

The Cochrane Q test and I^2^ statistic were adopted to evaluate heterogeneity among the studies. The choice of meta-analytic model was based on the I^2^ value: a random-effects model was used when I^2^ exceeded 50% and a fixed-effects model was used when I^2^ was below 50%. For outcomes with very low heterogeneity and a small number of contributing studies, use of a fixed-effect model was deemed applicable because estimating between-study variance in a random-effects model would have been unreliable. Forest plots were generated to graphically depict the direction and magnitude of the intervention effects across studies. Sensitivity analyses to assess robustness of pooled estimates by excluding high risk-of-bias studies were contingent on having at least two studies per outcome. Subgroup analyses were planned to explore heterogeneity and were conducted only when a minimum of two studies were available within a subgroup. Egger’s regression intercept was used to detect the presence of publication bias.

## 3. Results

### 3.1. Study Selection

The literature search yielded 856 potentially relevant articles along with two additional records that were identified through a citation search from the reference list of the eligible articles and were included in the pool of articles for consideration. After removing 283 duplicates, 573 papers were screened, and 55 full-text articles were reviewed to determine eligibility. Ultimately, 11 articles were included in the final review, out of which 8 possessed the required data quality to be subjected to meta-analysis. The complete search process with detailed representation of the review workflow is outlined in Figure 1. Throughout the different stages of screening and quality appraisal, Cohen’s kappa coefficient was used to quantify inter-rater reliability, which showed an overall excellent agreement between the two reviewers with scores of 0.98, 0.86, and 0.98, respectively.

### 3.2. Study Characteristics

Key features of the selected studies are shown in Table 1.

Eleven RCTs with 1000 participants were included in the review. Most studies were from Asia, particularly China (six) [28,29,30,31,32,35], and the rest were from Thailand (one) [36], Japan (one) [37], France (one) [38], Finland (one) [39], and Egypt (one) [40]. Sample sizes ranged from 50 to 180, with varying follow-up durations from 6 weeks to 3 years. The effect on nutritional status was measured via several subjective (PG-SGA and NRS 2002) and objective indicators such as anthropometric measures, body composition, and biochemical markers. Across the included studies, the most reported outcome was body weight [28,29,30,31,32,36,39], measured using standard calibrated weighing scales. Three studies [29,31,39] assessed body composition parameters, like fat-free mass (FFM), fat-free mass index (FFMI), body cell mass (BCM), skeletal muscle mass (SM), and phase angle (PA), using bioelectrical impedance analysis (BIA). Several studies evaluated patient-reported outcomes, with a focus on mucositis, pain and opioid use, and quality of life (QoL).

Among the eleven studies, concurrent chemoradiotherapy (CCRT) was administered in eight [28,29,30,31,36,37,38,39], while only three [32,35,40] used radiotherapy (RT) as the sole treatment modality.

### 3.3. Effects on Nutritional Outcomes

#### 3.3.1. Anthropometric Measurements and Body Composition

**Figure 2:**
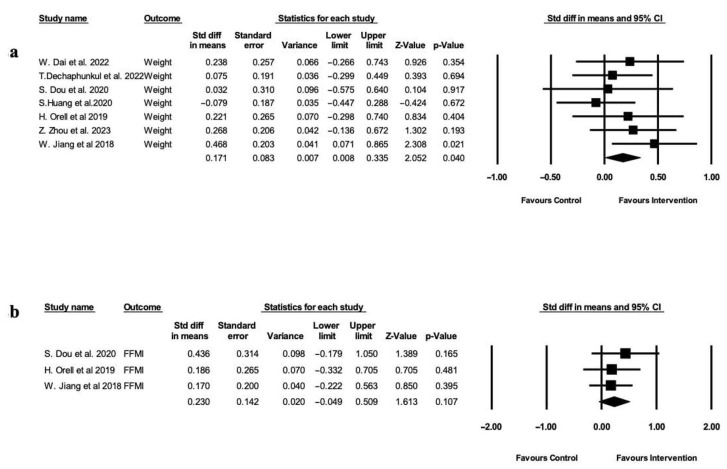
Forest plot depicting changes in nutritional status shown in standard difference in means with standard error compared to control group. (**a**) Body weight (kg). Heterogeneity: Tau^2^ = 0.000, Q = 4.711 (df = 6, *p* = 0.04), and I^2^ = 0.00%. (**b**) Fat-free mass index (FFMI) (kg/m^2^). Heterogeneity: Tau^2^ = 0.000, Q= 0.545 (df = 2, *p* = −0.107), and I^2^ = 0.00% [28,29,30,31,32,36,39].

Seven studies [28,29,30,31,32,36,39] assessed the effect of nutritional intervention on weight maintenance. The results showed a small but statistically significant (*p* =0.040) improvement in the intervention group, with a standardized mean difference (SMD) of 0.171 (95% CI: 0.008 to 0.335) (Figure 2a). Although the meta-analysis for the fat-free mass index (FFMI) (Figure 2b) using a fixed-effect model did not yield statistically significant results (95% CI: −0.049 to 0.509; *p* = 0.107), the pooled effect size of 0.230 hints at a possible benefit of nutritional intervention in preserving lean muscle mass and body composition in HNC patients during CRT. Marginal improvements in body cell mass (BCM) (*p* = 0.061) and phase angle (PA) (*p* = 0.074), were observed by Dou et al. [29], though these findings were not supported by other studies. To explore these trends and establish statistical significance, studies with a bigger sample size and extensive follow-up are needed.

#### 3.3.2. Biochemical Markers

**Figure 3:**
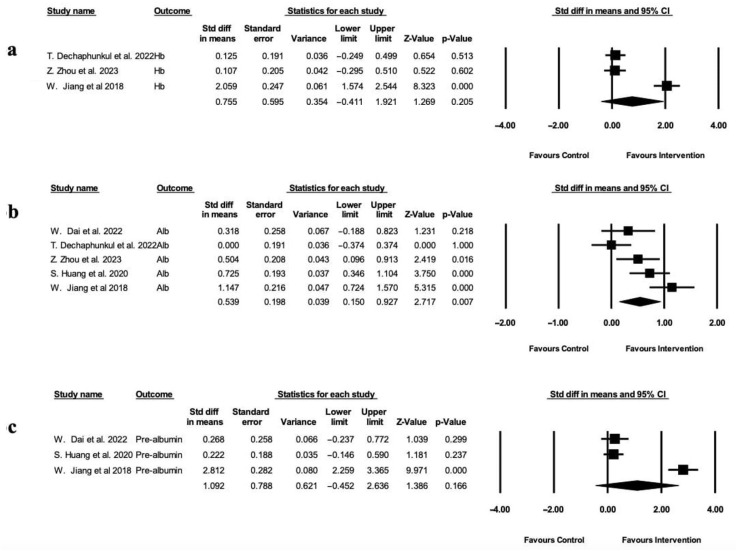
Forest plot depicting changes in biochemical markers shown in standard difference in means with standard error compared to control group. (**a**) Hemoglobin levels (g/dl). Heterogeneity: Tau^2^ = 1.015, Q-value = 46.725, and df = 2 (*p*-value = 0.205), I^2^ = 95.72%. (**b**) Albumin (g/L). Heterogeneity: Tau^2^ = 0.151, Q-value = 17.614, df = 4 (*p*-value = 0.007), and I^2^ = 77.29%. (**c**) Pre-albumin levels (mg/L). Heterogeneity: Tau^2^ = 1.802, Q-value = 64.614, df = 2 (*p*-value = 0.166), and I^2^ = 96.91% [28,30,31,32,36].

Random-effect analysis of three studies [31,32,36] revealed a favorable but non-significant effect of nutritional intervention on hemoglobin levels (SMD = 0.755; 95% CI: −0.411 to 1.921; *p* = 0.205) (Figure 3a), with high heterogeneity (I^2^ = 95.72%). While Jiang et al. [31] reported a large and significant rise in hemoglobin levels in the intervention group, the other two studies found only negligible effects. The meta-analysis of five studies [28,30,31,32,36] demonstrated a statistically significant improvement in serum albumin levels in the intervention group (SMD = 0.539; 95% CI: 0.150 to 0.927; *p* = 0.007) (Figure 3b). The effect of nutrition-based interventions on serum pre-albumin levels was evaluated in three studies [28,30,31] (Figure 3c). Although the meta-analysis using random-effects favored the intervention group, it yielded a non-significant pooled estimate (SMD = 1.092; 95% CI: −0.452 to 2.636; *p* = 0.166) and was associated with high heterogeneity (I^2^ = 96.90%). The result was largely driven by the substantial effect size (SMD = 2.812; *p* < 0.001) reported in Jiang et al. [31], in contrast to the smaller, non-significant findings seen in the other two studies [28,30]. Two studies [31,39] narratively reported higher transferrin levels in the study groups; however, statistically relevant inter-group differences were not seen.

#### 3.3.3. PG-SGA Score

**Figure 4:**
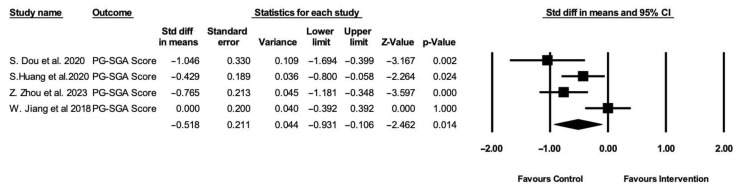
Forest plot depicting changes in PG-SGA score shown in standard difference in means with standard error compared to control group. Heterogeneity: Tau^2^ = 0.124, Q = 10.517 (df = 3, *p* = 0.014), and I^2^ = 71.47% [29,30,31,32].

In addition to objective indicators, subjective nutritional status was assessed in multiple studies using the patient-generated subjective global assessment (PG-SGA) tool. The pooled analysis of four studies showed significant improvements in nutritional status in the intervention group (SMD = −0.518 95% CI: −0.931 to −0.106, *p* = 0.014) (Figure 4). A negative effect size reflects a lower PG-SGA score in the study group, suggesting improved nutritional status.

#### 3.3.4. Energy and Protein Intake

Three studies [28,35,39] examined changes in energy and protein intake using dietary assessment tools. One study investigating the role of NC employed weekly through 24 h dietary recalls and demonstrated significantly greater calorie and protein consumption in the intervention group from 4th week of CRT onwards [28]. Orell et al. [39] used food diaries and dietary recall to measure nutritional adequacy relative to estimated needs but found no significant between-group differences by the end of the treatment. The third study calculated daily energy and protein intake against pre-defined targets (6–7 kJ/kg/day and 1.2–1.5 g/kg/day) but failed to provide comparative outcome data across the groups. Even though there is a suggested increase in dietary intake via some nutritional interventions, evidence remains inconclusive due to lack of standardized measurement tools and reporting completeness.

### 3.4. Effects on Clinical and Patient-Reported Outcomes

#### 3.4.1. Oral Mucositis

Despite the incidence and severity of oral mucositis being commonly evaluated across the included studies, quantitative synthesis could not be carried out due to methodological heterogeneity in grading systems, time points, and outcome reporting (severity vs. grade of mucositis). While two trials reported a significant benefit of immunonutrition in reducing the severity of radiation-induced mucositis [37,40], two others using similar interventions observed no such significant difference between the groups [36,38]. These conflicting findings highlight the variability in outcomes and underscore the need for further investigation.

#### 3.4.2. Pain and Opioid Use

One study measured pain levels using the Visual Analog Scale (VAS) and reported significantly lower scores in the intervention group, along with a reduced need for opioids in those receiving immunonutrition [40]. In contrast, Kuroki et al., using a similar nutritional intervention, found no significant difference in opioid use among the study arms [37].

#### 3.4.3. Quality of Life

Contrasting findings were reported in two studies using the EORTC QLQ-C30 to evaluate the impact of dietary intervention on QoL. While Zhu et al. [35], who examined the combined effect of swallowing rehabilitation and nutritional support, reported a statistically significant improvement in global QoL outcomes in the intervention arm, three months post-radiotherapy (*p* = 0.044), S. Huang et al. [30] found no significant changes in QoL scores between the groups at the end of CRT after adjusting for baseline values, despite slightly higher scores in the control group. Similarly, another study investigating the effect of ONS during treatment showed a decline in QoL scores across several domains in each group, with follow-up assessments revealing no significant inter-group differences. Due to inconsistent reporting formats, variation in follow-up duration, and the limited number of studies, a meta-analysis for this outcome could not be conducted.

#### 3.4.4. Psychological Indicators

Only one study [28] used a standardized tool (Hospital Anxiety and Depression Scale) to assess psychological well-being and reported significantly lower anxiety and depression scores in the intervention group during treatment. The remaining studies did not employ validated measures of mental health, precluding further synthesis of psychological outcomes.

### 3.5. Quality Assessment

Seventy-three percent of the selected studies demonstrated an elevated bias risk due to inadequate blinding, mainly because of the inherent difficulty in masking nutritional interventions in oncology settings [41]. Only 36% adequately described allocation concealment and over 50% reported complete follow-up, with the remainder lacking sufficient details to rule out potential selection and attrition bias. These quality concerns reflect methodological limitations, which are further elaborated in this discussion and should be considered while interpreting the results of this study. The result for the quality assessment is included in the Supplementary Materials (Figure S1). After applying the GRADE approach, the strength of the evidence across the outcomes varied from moderate to very low (for albumin, hemoglobin, pre-albumin, and PG-SGA), details of which are presented in the Summary of Findings (SoF) table (Table 2).

### 3.6. Sensitivity Analysis

Sensitivity analysis excluding studies with a high risk of bias was attempted. However, the exclusion of high-risk studies left only one low-risk trial contributing data for key outcomes, thus preventing a meaningful re-estimation of the pooled effects and making sensitivity analysis statistically infeasible.

### 3.7. Subgroup Analysis

A subgroup analysis was planned to explore the potential sources of heterogeneity observed for certain outcomes. Studies were categorized by intervention type (ONS, nutritional counseling, and immunonutrition). For hemoglobin, although three studies [31,32,36] contributed data, each belonged to a different intervention category, making pooled subgroup meta-analysis statistically infeasible and accounting for the very-high heterogeneity observed in the overall analysis (I^2^ = 95.7%). Similarly for pre-albumin, studies using ONS as intervention [30,31] showed a large, pooled effect (SMD = 1.408; 95% CI 0.149–2.668) but with very high heterogeneity (I^2^ = 89.5%). Only one study [28] reported a small, non-significant effect (SMD = 0.268; 95% CI −0.237–0.772) with nutritional counseling. Substantial between-study differences in effect size (ranging from SMD ≈ 0.22 to 2.81) and differences in intervention type are likely responsible for the high heterogeneity observed in the overall analysis.

### 3.8. Publication Bias

Examination of the funnel plots revealed no significant asymmetry, and Egger’s regression test with *p*-values for all parameters greater than 0.05 suggested an absence of statistically significant publication bias. The funnel plots for all parameters are included in the Supplementary Materials (Figures S2-1 and S2-2).

## 4. Discussion

This study synthesized evidence from 11 RCTs evaluating the impact of dietary interventions on nutritional status, energy and protein intake, and clinical and patient-reported outcomes in HNC patients undergoing CCRT. The main findings indicated that non-invasive, patient-directed nutritional interventions, including oral nutritional supplements, dietary counseling, and immune-enhanced nutrition, significantly improved weight maintenance, serum albumin levels, and subjective nutritional status (PG-SGA scores). The direction of effect consistently favored the intervention group, suggesting clinically meaningful benefits.

Nutritional interventions positively influenced weight maintenance, marked by a small but statistically significant pooled effect size (SMD = 0.171), which was notable given the well-recognized risk of severe weight loss among HNC patients during CRT [42]. FFM and FFMI are reflectors of body composition and prognostic markers of oncological outcomes in HNC patients [43]. The results of this study align with the existing literature [44] showing a non-significant trend in FFMI favoring the intervention group. The lack of significance could be attributed to extensive systemic inflammation and hyper catabolism induced by intensive chemoradiotherapy, which may not be fully mitigated by nutritional support alone [45,46].

Among the biochemical markers, serum albumin alone showed a statistically significant improvement, which is clinically relevant given its dual role as an indicator of both nutritional status and inflammatory response [47]. This improvement in contrast to hemoglobin and pre-albumin which did not show significant changes—likely due to their sensitivity to inflammation, shorter half-lives, and tendency to be influenced by factors beyond nutrition alone, as noted in previous studies [48], highlights the complexity of interpreting biochemical responses to nutritional interventions in cancer care settings. Future research should consider longer intervention durations, targeted micronutrient supplementation, and concurrent monitoring of inflammatory markers to better capture changes in these biomarkers, thereby increasing the validity and interpretability of biochemical outcomes.

The differential effect of nutritional interventions observed across studies can be attributed to the type, timing of intervention initiation, and underlying patient characteristics. For example, a study [30] delivering prophylactic ONS documented fewer treatment interruptions, in contrast to the greater symptom burden and lower adherence rates observed in studies where nutritional support was reactively administered [39]. Similar findings are reported in other studies, where a higher completion rate of concurrent chemotherapy and reduced suspension or delays in radiotherapy were observed with prophylactic ONS, thus highlighting the importance of instituting nutritional interventions early in the treatment trajectory before deterioration sets in [49]. Another factor possibly affecting the study outcomes was compliance with the prescribed intervention. Higher compliance was explicitly associated with better clinical and nutritional outcomes, highlighting the critical role of compliance in determining the effectiveness of nutritional interventions [29,31,38,39]. However, it waned across studies when patients encountered treatment-related toxicities, suggesting the difficulty in maintaining prescribed intake under treatment-related stress. Evidence supporting consistent adherence to nutritional interventions remains scarce. Tailoring interventions to patient preferences and integrating behavioral support strategies may be associated with sustained adherence and improved clinical outcomes [50].

Across the studies included, QoL and psychological indicators remained under-reported. QoL was assessed using different measurement tools capturing various domains, limiting cross-study comparability and making it difficult to draw meaningful conclusions. Given the known links between nutritional status and psychological distress, future studies should routinely include validated QoL and psychological measures to capture the broader effect of nutritional interventions [51,52].

### 4.1. Future Directions

This review highlights key priorities for advancing nutritional care in head and neck cancer patients undergoing chemoradiotherapy. Future studies should prioritize large, multicentre randomized controlled trials with standardized nutritional protocols and harmonized outcome measures to overcome the limitations of small, single-center designs. Strategies to improve patient adherence, including behavioral support and technology-based monitoring, should be systematically evaluated, and longer follow-up is essential to clarify the long-term effects of nutritional interventions on survival, treatment tolerance, and recovery. In addition, research exploring personalized approaches tailored to baseline nutritional status, integration with multidisciplinary supportive care, and cost-effectiveness analysis will provide valuable insights for clinical implementation.

### 4.2. Strengths and Limitations

This systematic review and meta-analysis is strengthened by its adherence to PRISMA guidelines, adoption of a through and systematic search, and inclusion of only RCTs to enhance methodological rigor [53]. Quantitative synthesis of outcomes using meta-analytic techniques was attempted to improve the robustness of the findings [54]. Despite these, certain limitations should also be acknowledged. Substantial risk of bias noted across the studies could have potentially compromised the internal validity and reliability of the pooled estimates. Evidence certainty for most outcomes, as appraised via the GRADE framework, was moderate to very low, which might limit the strength of the conclusions that can be drawn from this study. The presence of substantial heterogeneity in intervention types, measurement tools, time points of evaluation, and follow-up durations complicated data synthesis and the interpretation of effect estimates. The relatively short follow-up durations employed in most trials restricted evaluation of the long-term effects of nutritional interventions on clinical and patient-reported outcomes. Furthermore, several studies with smaller sample sizes reduced the statistical power for subgroup or sensitivity analysis. In particular, the number of studies with a low risk of bias was insufficient to conduct sensitivity analysis excluding the high-risk studies for any outcome. Although subgroup analysis was conducted based on intervention type, the small number of studies within each subgroup limits the interpretability and strength of these findings, warranting a cautious interpretation of the pooled estimates. Finally, the predominance of studies from China (n = 6) may restrict the generalizability of our findings to wider settings.

### 4.3. Conclusions

This study highlights the potential of nutritional interventions as critical adjuncts to standard oncologic care for patients with head and neck carcinoma receiving concurrent chemoradiotherapy. These interventions included oral nutritional supplements, dietary counseling, and immune-enhanced nutrition. While the magnitude of interventional effects varied, consistent trends favoring intervention were observed for weight maintenance, serum albumin levels, and subjective nutritional status. However, evidence remains limited or inconclusive for outcomes such as hemoglobin, pre-albumin, quality of life, and symptom relief. By systematically synthesizing data across nutritional, biochemical, and patient-reported outcomes, this review addresses a key gap in the existing literature on non-invasive, patient-directed nutritional strategies in HNC care. The findings underscore the urgent need for well-designed randomized controlled trials employing standardized intervention protocols, consistent outcome reporting, longer follow-up periods, and broader psychosocial assessments in the future. Strengthening the evidence base is essential for developing research-informed nutritional guidelines and supporting individualized care pathways aimed at optimizing therapeutic tolerability, quality of life, and overall outcomes in the HNC population.

## Figures and Tables

**Table 1:** Characteristics of the included studies.

Author/ Year Country	Sample Size	Cancer Stage/Sub Type	Treatment Received	Intervention Type	Follow-Up Period	Outcomes Measured	Tools for Measurement of Outcomes	Main Findings
W. Dai et al./2022 [28] China	72 (ITT) (IG: 36; CG: 36) 61(PP) IG: 32 CG: 29	Stage III/IV HNC	IMRT + Concurrent CT	IG: NC by RD CG: Standard care based on clinician’s experience	6-week follow-up during CCRT (evaluations at baseline, 2, 4, and 6 weeks)	Body weight TSF Calorie and protein intake S total protein, albumin, transferrin, and pre-albumin KPS Anxiety and depression	Weighing scale, skinfold caliper, 24 h dietary recall, and blood tests KPS Scale HADS-A and HADS-D questionnaire	Less weight loss in IG, less reduction in TSF thickness. Decreased in both groups but significantly better maintained in IG. The biochemical markers showed significantly lower decline in IG. Higher scores on KPS scale showing better functional status. Significant reduction in anxiety and depression level.
S. Dou et al./2020 [29] China	52 (ITT) 42 (PP) IG: 23 CG:19	Stage II-IVb NPC	CCRT	IG: Individualized dietary counseling along with ONS CG: Individualized dietary counseling	6-week follow-up during CCRT (evaluations at baseline, 2, 4, 6 weeks)	Body weight and BMI FFMI, SM, BCM, and PA Nutritional status Treatment-related toxicity	Weighing scale BIA PG-SGA [30] CTCAE v3.0	Decreased in both groups; slightly better maintenance with >2/3 ONS intake (*p* = 0.170) Significantly better in ONS >2/3 group. PG-SGA scores better in ONS group at the end of treatment (*p* = 0.053). No significant difference in Grade III–IV mucositis between groups
S. Huang et al./2020 [30] China	114 (ITT) IG: 58 CG: 56	Stage III-IV NPC	Neo-adjuvant chemotherapy followed by CCRT	IG: Prophylactic ONS with dietitian support CG: Regular diet; ONS provided only if clinically necessary (e.g., weight loss > 5%, intake < 50%)	From baseline through 3 months after radiotherapy (6 time points: T1–T6)	Weight loss (%) PG-SGA [30] NRS-2002 [31] score (to assess nutritional status), serum albumin, prealbumin, and total protein QoL CT/RT interruptions	Weighing scale, PG-SGA, and NRS-2002 Scale Blood tests EORTC QLQ-C30 Medical records	Decreased over time with no significant difference in weight loss, nutritional status, QoL, and global health status between two groups. Pre-albumin shows no significant difference (0.73). Total protein is significantly better in CG (*p* = 0.008). Better treatment tolerance: lower RT interruption (0% vs. 7.14%) and chemotherapy interruption (10.34% vs. 28.57%) in intervention group.
Z. Zhou et al./2023 [32] China	116 (ITT) IG: 61 CG: 55 95 (PP) IG: 48 CG: 47	HNC/Esophageal cancer	IMRT	IG: HCF nutritional management model including weekly follow-up, community doctor support, and an individualized nutrition plan based on PG-SGA CG: Conventional hospital-based nutrition management only	Weekly evaluations for 3 months after EoRT	PG-SGA [33] and NRS2002 [34] score ECOG performance score Weight loss Serum albumin, prealbumin, and hemoglobin Treatment-related toxicity	PG-SGA [33] and NRS2002 [34] scoring system ECOG performance scale Weighing scale Blood tests RTOG grading for radiation toxicity	HCF group showed significantly better PG-SGA (*p* < 0.001), NRS2002 (*p* < 0.001), ECOG (*p* = 0.006), and less weight loss (*p* = 0.024). Higher albumin (*p* = 0.001), prealbumin (*p* = 0.046), and hemoglobin (*p* = 0.013). Reduced severity of radiation mucositis (*p* = 0.018) and dermatitis (*p* = 0.028).
W. Jiang et al./2018 [31] China	100 (ITT) IG: 50 CG: 50 95 (PP)	Stage III–IV NPC	Induction chemotherapy before CRT	IG: ONS + Regular meals CG: Regular diet + general dietary advice (no ONS)	From baseline to end of CCRT, and 3-month post-CRT follow-up	Body weight, BMI, FFM, FFMI, hemoglobin, albumin, and prealbumin, transferrin PG-SGA score QoL Treatment tolerance and CRT-related toxicities	PG-SGA EORTC QLQ-C30 and H&N35 Lab tests	ONS group had significantly higher body weight (*p* = 0.036), BMI (*p* = 0.021), and prealbumin (*p* = 0.048) at the end of CRT. No significant difference in PG-SGA, FFM, FFMI, or QoL between groups. No major difference in CRT-related toxicity; compliance fair, but some nausea reported.
X. Zhu et al./2022 [35] China	66 IG: 33 CG: 33	Laryngeal cancer and dysphagia post-total laryngectomy and radiotherapy	RT	IG: Swallowing training + individualized nutritional intervention (weekly sessions with a dietitian for 3 months, meal planning with caloric/protein goals, and ONS/tube feeding if needed) CG: Routine health counseling + swallowing training only	3 months post-RT	Swallowing function Nutritional status QoL	VFSE PG-SGA scale EORTC QLQ-C30 questionnaire	Both groups improved in VFSE, PG-SGA, and QLQ-C30 scores, but the intervention group improved significantly more. - VFSE: 3.64 vs. 2.23 (*p* = 0.013); - PG-SGA: 1.92 vs. 0.69 (*p* = 0.002); - QLQ-C30: 8.28 vs. 4.45 (*p* = 0.044).
T. Dechaphunkul et al./2022 [36] Thailand	110 (ITT) IG: 55 CG: 55	Stage II–IVb non-metastatic HNC	CCRT	IG: Immune nutrient formula containing omega-3 fatty acids, arginine, dietary nucleotides, and soluble fiber. (For 5 days before each CT cycle) CG: Isocaloric isonitrogenous standard enteral nutrition formula	Median 42.2 months; assessments during treatment and up to 3 years for survival	Severe OM (grade 3–4) Treatment-related toxicities Body weight Serum albumin Inflammatory markers (CRP, NLR, and PLR) PFS, OS	CTCAE v4.03 Weight scale Blood tests Kaplan–Meier for survival analyses	No significant difference in OM (62% vs. 67%, *p* = 0.690). No differences in weight loss or serum markers. PFS at 3 years: 69% (IG) vs. 44% (CG), *p* = 0.056. OS at 3 years: 69% vs. 50%, *p* = 0.065 (not statistically significant). More treatment delays in CG (56% vs. 38%).
K. Kuroki et al./2023 [37] Japan	75 (ITT) IG: 38 CG: 37 58 (PP) IG: 24 CG: 34	Non-metastatic HNC	PBCRT	IG: Immunonutrition containing HMB/Arg/Gln CG: Not given	Weekly assessments during CRT and two weeks post-treatment	Incidence of Grade ≥ 2 and ≥3 mucositis Body weight Opioid use CRT suspension duration	CTCAE v4.03 Weighing scale Medical records	Grade 3 mucositis: 25% (IG) vs. 64.6% (CG), *p* = 0.0037. Weight loss: 3.57 kg (5.6%) in IG vs. 5.89 kg (8.9%) in CG, *p* = 0.0020/0.0038. No significant difference in opioid use (25% vs. 35.3%). Shorter CRT delays in IG, though not statistically significant.
P. Boisselier et al./2020 [38] France	180 (ITT) IG: 90 CG: 90 172 (PP) IG: 86 CG: 86	HNC	CCRT post-surgery	IG: Immunonutrition enriched with l-arginine, omega-3 fatty acids, and RNA CG: Isocaloric isonitrogenous formula	Up to 3 years of assessments weekly during CRT, 1-month post-CRT, and annual survival follow-ups	Grade 3–4 OM Treatment-related toxicities, and CT interruptions Compliance OS and PFS	RTOG and WHO grading CTCAE v4.03 Compliance via sachet count and patient reporting Kaplan–Meier and log-rank tests	No significant difference in grade 3–4 mucositis between groups. No differences in toxicity or chemo delays overall. In ≥75% compliant patients: OS at 3 years was significantly better (81% vs. 61%, *p* = 0.034); PFS also improved (73% vs. 50%, *p* = 0.012).
H. Orell et al./2019 [39] Finland	65 (ITT) IG: 32 CG: 33 58 (PP) IG: 26 CG: 32	Stage III–IV HNC	CCRT	IG: Intensive nutritional counseling (INC) by a dietitian CG: On-demand counseling (ODC) by a dietitian	During CRT (6–7 weeks) and survival follow-up for median 43 months	Nutritional status Body weight, BMI, FFM, FFMI, fat mass, and Handgrip strength (HGS) Nutritional intake (kcal/kg, protein intake) Treatment-related toxicities, mucositis, and nausea Survival (OS, DFS, and DSS)	PG-SGA Weighing scale BIA JAMAR dynamometer Dietary recall and PEG feed tracking CTCAE v3.0 Kaplan–Meier/log-rank	No significant difference between two groups for nutritional status or outcomes. A total of 71% experienced critical weight loss (>5%). Pre-treatment malnutrition and low HGS predicted poorer survival (OS and DFS). INC did not significantly reduce mucositis or weight loss compared to ODC. Many patients (69%) struggled to complete planned nutritional treatment.
S. S. Ibrahim et al./2024 [40] Egypt	50 (ITT) IG: 25 CG: 25 40 (PP) IG: 20 CG: 20	Stage I–IV HNC	RT	IG: Single agent immunonutrition (Oral L-glutamine suspension (5 g glutamine + 5 g maltodextrin in water) CG: Placebo suspension of 10 g of maltodextrin dissolved in cold water	From baseline (RIOM onset) to EoRT (5–7 weeks); 3 assessments: baseline, 2 weeks, end of RT	OM Pain Opioid usage incidence BMI Salivary TGF-β1 levels	WHO Oral Toxicity Scale OMAS VAS kg/m^2^ ELISA	Significant reduction in mucositis severity in IG on WHO and OMAS (*p* < 0.001). Pain-VAS scores and opioid use significantly reduced (*p* < 0.001 and *p* = 0.014). BMI more stable in glutamine group; significant intergroup difference in BMI change (*p* < 0.001). Salivary TGF-β1 decreased in IG, increased in CG *p* < 0.001). Strong correlations between OM severity and TGF-β1 (r = 0.738), pain (r = 0.737), and BMI (r = –0.899).

RT: radiotherapy; IMRT: intensity-modulated radiotherapy; CT: chemotherapy; CRT: chemoradiotherapy; CCRT: concurrent chemoradiotherapy; IG: intervention group; CG: control group; NC: nutritional counseling; ONS: oral nutritional supplements; RD: registered dietitian; TSF: triceps skin fold; FFM: fat-free mass; FFMI: fat-free mass index; OM: oral mucositis; QoL: quality of life; BMI: body mass index; OS: overall survival; PFS: progression-free survival; NRS: nutritional risk screening; RTOG: Radiation Therapy Oncology Group; CTCAE: Common Terminology Criteria for Adverse Events; ECOG: Eastern Cooperative Oncology Group; PG-SGA: Patient Generated Subjected Global Assessment; EORTC QLQ-C30: European Organization for Research and Treatment of Cancer Quality of Life Core Questionnaire; EoRT: end of radiotherapy.

**Table 2:** Grade quality assessment for included studies in the meta-analysis.

		Certainty Assessment								
Outcomes	n	Study Design	RoB	Inconsistency	Indirectness	Imprecision	Other Considerations	No. of Participants	Effects	Certainty
Weight maintenance	7	RCT	Not serious	Not serious	Not serious	Not serious	none	622	SMD = 0.171 (95% CI: 0.008 to 0.335), *p* = 0.040	Moderate
FFMI	3	RCT	Serious	No serious	No serious	Serious (small sample size, CI crosses 0)	none	217	SMD = 0.230 (95% CI: –0.049 to 0.509), *p* = 0.107	Low
Albumin	5	RCT	Serious	Serious	Serious	No serious	none	512	SMD = 0.539 (95% CI: 0.150 to 0.927), *p* = 0.007	Very Low
Hemoglobin	3	RCT	Serious	Very Serious (very high heterogeneity)	Serious	Serious (small sample, wide CI)	none	326	SMD = 0.755 (95% CI: –0.411 to 1.921), *p* = 0.205	Very Low
Pre-albumin		3 RCT	Serious	Serious (very high heterogeneity)	Not serious	Serious (small sample size, wide CI, effect driven by single study)	none	314	SMD = 1.092 (95% CI −0.452 to 2.636), *p* = 0.166	Very low
PG-SGA	4	RCT	Serious	Serious	Not serious	Serious	none	382	SMD = –0.518 (95% CI: –0.931 to –0.106), *p* = 0.014	Very low
OM	4	RCT	Serious (narrative synthesis)	Serious (inconsistent findings)	Serious (different grading systems)	Serious (small sample size)	none	234	Not pooled (Narrative synthesis)	Very low
QoL	3	RCT	Serious (methodological limitations)	Serious (inconsistent outcomes)	Serious (different measurement tools)	Serious (small sample size)	none	202	Not pooled (Narrative synthesis)	Very low

Abbreviations. FFMI: fat-free mass index, PG-SGA: patient-generated subjective global assessment, OM: oral mucositis. QoL: quality of life. [Downgraded for RoB due to allocation concealment; downgraded for inconsistency due to high heterogeneity; downgraded for indirectness because albumin/Hb are non-specific nutrition markers].

## Data Availability

No new data were created or analyzed in this study. Data sharing is not applicable to this article.

## Supplementary Materials

The following supporting information can be downloaded at: https://www.mdpi.com/article/10.3390/healthcare13243324/s1. Supplementary Table S1: Search Strategy. Supplementary Table S2: Grade Quality assessment for included studies of the meta-analysis. Supplementary Figure S1: Risk of bias summary for the included studies, based on JBI Checklist for RCTs, visualized using robvis traffic-light plot, with custom mapping for each checklist item. Supplementary Figure S2-1: Funnel Plots of Included Studies (a. weight; b. FFMS; c. PG-SGA). Supplementary Figure S2-2: Funnel Plots of Included Studies (d. hemoglobin; e. albumin; f. pre-albumin).
