# Supplementary material for: Nutritional Interventions in Head and Neck Cancer Patients Undergoing Chemoradiotherapy: A Systematic Review and Meta-Analysis
Source: Healthcare (Basel). 2025 Dec 18;13(24):3324. doi: 10.3390/healthcare13243324 (PMC12732905; doi:10.3390/healthcare13243324)
Supplement: Supplementary file 1 [file healthcare-13-03324-s001.zip › healthcare-3943295-supplementary.pdf]

Supplementary Table S1: Search Strategy

**Search terms for PUBMED**
(("Head and Neck Neoplasms"[MeSH] OR "Oral Neoplasms"[MeSH] OR "Laryngeal Neoplasms"[MeSH] OR<br>"Pharyngeal Neoplasms"[MeSH] OR "Nasopharyngeal Neoplasms"[MeSH] OR<br>"head and neck cancer"[tiab] OR "oral cancer"[tiab] OR "laryngeal cancer"[tiab] OR<br>"pharyngeal cancer"[tiab] OR "nasopharyngeal cancer"[tiab])<br>**AND**<br>("Nutrition Therapy"[MeSH] OR "Diet Therapy"[MeSH] OR "Nutritional Support"[MeSH] OR<br>"oral nutritional supplement"[tiab] OR "nutritional intervention"[tiab] OR "nutritional support"[tiab] OR<br>"ONS"[tiab] OR "immunonutrition"[tiab] OR "omega 3 fatty acids"[tiab] OR "arginine"[tiab] OR<br>"glutamine"[tiab])<br>**AND**<br>("Body Composition"[MeSH] OR "Nutritional Status"[MeSH] OR "Quality of Life"[MeSH] OR<br>"biochemical markers"[tiab] OR "inflammatory markers"[tiab] OR "albumin"[tiab] OR "prealbumin"[tiab] OR<br>"weight loss"[tiab] OR "BMI"[tiab] OR "QoL"[tiab]))<br><br>Filters: Humans, English, Randomized Controlled Trials, 2019/01/01–2024/09/15
**Search terms for CINAHL (EBSCOhost)**
((MH "Head and Neck Neoplasms+" OR MH "Oral Neoplasms+" OR MH "Laryngeal Neoplasms+" OR MH "Pharyngeal Neoplasms+" OR MH "Nasopharyngeal Neoplasms+")<br>OR(TX "head and neck cancer*" OR "head and neck tumor*" OR "head and neck carcinoma*" OR "head and neck malignanc*" OR "head and neck neoplasm*"OR "oral cancer*" OR "oral carcinoma*" OR "oral malignanc*" OR "oral tumor*" OR "oral cavity cancer*" OR "oral cavity neoplasm*"OR "laryngeal tumor*" OR "laryngeal cancer*" OR "laryngeal carcinoma*" OR "laryngeal malignanc*" OR "laryngeal neoplasm*"<br>OR "pharyngeal tumor*" OR "pharyngeal cancer*" OR "pharyngeal carcinoma*" OR "pharyngeal malignanc*" OR "pharyngeal neoplasm*"OR "nasopharyngeal carcinoma*" OR "nasopharyngeal tumor*" OR "nasopharyngeal cancer*" OR "nasopharyngeal malignanc*"))<br>**AND**<br>((MH "Nutrition Therapy+" OR MH "Nutritional Support" OR MH "Diet Therapy" OR MH "Dietary Supplements") OR (TX "nutrition*" OR "nutritional intervention*" OR "nutritional support" OR "nutritional advice" OR "nutritional therap*" OR "nutritional recommendation*OR "dietary therapy" OR "dietary support" OR "dietary intervention*" OR "dietary advice" OR "dietary recommendation*" OR "nutritional counseling"

OR "dietary counseling" OR "oral nutritional supplement*" OR "nutritional supplement*" OR "ONS" OR "immunonutrition"OR "immune enhancing nutrition" OR "immune modulating nutrition" OR "arginine" OR "glutamine" OR "omega 3 fatty acid*" OR "nucleotide*" OR "immunonutrient enriched formula" OR "amino acid*" OR "branched chain amino acid*" OR "polyunsaturated fatty acid enriched supplement*"OR "high energy ONS" OR "high protein ONS"))   **AND**   ((MH "Body Composition" OR MH "Nutritional Status" OR MH "Quality of Life" OR MH "Biological Markers" OR MH "Inflammation" OR MH "Toxicity") OR   (TX "body composition" OR "weight change*" OR "body weight" OR "weight loss" OR "BMI" OR "nutritional status" OR "nutritional intake" OR "treatment related toxicit*" OR "treatment related side effect*" OR "chemoradiation related toxicit*" OR "radiotherapy related toxicit*"   OR "biochemical parameter*" OR "biochemical marker*" OR "inflammatory marker*" OR "inflammatory mediator*" OR "laboratory parameter*"OR "hematological parameter*" OR "CRP" OR "hemoglobin" OR "albumin" OR "prealbumin" OR "transferrin" OR "TLC" OR "WBC count"OR "quality of life" OR "health related quality of life" OR "QoL" OR "well-being"))   **AND**   (RCT* OR "random* control* trial*" OR "control* clinical trial*" OR "control* trial" OR "random* control* stud*" OR "control* stud*" OR "control* clinical stud*") **OR** ((MH "Randomized Controlled Trials as topic+") OR (MH "Controlled Clinical Trials as Topic+")) <br>Filters: Humans, English, 2019/01/01–2024/09/15
**Search terms for SCOPUS**
TITLE-ABS-KEY(("head and neck cancer*" OR "head and neck tumor*" OR "head and neck carcinoma*" OR "head and neck malignanc*" OR "head and neck neoplasm*"OR "oral cancer*" OR "oral carcinoma*" OR "oral malignanc*" OR "oral tumor*" OR "oral cavity cancer*" OR "oral cavity neoplasm*"OR "laryngeal tumor*" OR "laryngeal cancer*" OR "laryngeal carcinoma*" OR "laryngeal malignanc*" OR "laryngeal neoplasm*"OR "pharyngeal tumor*" OR "pharyngeal cancer*" OR "pharyngeal carcinoma*" OR "pharyngeal malignanc*" OR "pharyngeal neoplasm*"OR "nasopharyngeal carcinoma*" OR "nasopharyngeal tumor*" OR "nasopharyngeal malignanc*" OR "nasopharyngeal cancer*")   **AND**   ("nutrition*" OR "nutritional intervention*" OR "nutritional support" OR "dietary therap*" OR "oral nutritional supplement*" OR "ONS" OR "immunonutrition" OR "arginine" OR "glutamine" OR "omega 3 fatty acid*" OR "amino acid*" OR "high protein ONS")   **AND**   ("body composition" OR "weight change*" OR "BMI" OR "nutritional status" OR "biochemical marker*" OR "albumin" OR "CRP"OR "hemoglobin" OR "quality of life" OR "QoL" OR "well-being")   **AND**

("random* control* trial*" OR "control* clinical trial*" OR "control* trial*" OR "random* control* stud*"OR "control* stud*" OR "control* clinical stud*" OR RCT*))<br>Filters: Humans, English, 2019/01/01–2024/09/15
**Search terms for ProQuest**
AB(("head and neck cancer*" OR "head and neck tumor*" OR "head and neck carcinoma*" OR "head and neck malignanc*" OR "head and neck neoplasm*"OR "oral cancer*" OR "oral carcinoma*" OR "oral malignanc*" OR "oral tumor*" OR "oral cavity cancer*" OR "oral cavity neoplasm*"OR "laryngeal tumor*" OR "laryngeal cancer*" OR "laryngeal carcinoma*" OR "laryngeal malignanc*" OR "laryngeal neoplasm*"OR "pharyngeal tumor*" OR "pharyngeal cancer*" OR "pharyngeal carcinoma*" OR "pharyngeal malignanc*" OR "pharyngeal neoplasm*"OR "nasopharyngeal carcinoma*" OR "nasopharyngeal tumor*" OR "nasopharyngeal malignanc*" OR "nasopharyngeal cancer*")<br>**AND**<br>("nutrition*" OR "nutritional intervention*" OR "nutritional advice" OR "nutritional support" OR "nutritional therap*" OR "nutritional recommendation*"OR "dietary therap*" OR "dietary support" OR "dietary intervention*" OR "dietary advice" OR "dietary recommendation*"<br>OR "nutritional counseling" OR "dietary counseling" OR "oral nutritional supplement*" OR "nutritional supplement*" OR "ONS"OR "immunonutrition" OR "immune enhancing nutrition" OR "immune modulating nutrition" OR "arginine" OR "glutamine" OR "omega 3 fatty acid*" OR "nucleotide*" OR "immunonutrient enriched formula" OR "amino acid*" OR "branched chain amino acid*"OR "polyunsaturated fatty acid enriched nutritional supplement*" OR "high energy ONS" OR "high protein ONS" OR "omega 3 supplement*")<br>**AND**<br>("body composition" OR "weight change*" OR "body weight" OR "BMI" OR "weight loss"<br>OR "nutritional status" OR "nutritional intake" OR "treatment related toxicit*" OR "treatment related side effect*" OR "chemoradiation related toxicit*" OR "radiotherapy related toxicit*"<br>OR "biochemical parameter*" OR "biochemical marker*" OR "inflammatory marker*" OR "inflammatory mediator*"OR "laboratory parameter*" OR "hematological parameter*" OR "CRP" OR "hemoglobin" OR "albumin" OR "prealbumin" OR "transferrin" OR "TLC" OR "WBC count" OR "quality of life" OR "health related quality of life" OR "QoL" OR "well-being")<br>AND<br>(RCT* OR "random* control* trial*" OR "control* clinical trial*" OR "control* trial*" OR "random* control* stud*" OR "control* stud*" OR "control* clinical stud*"))<br>Filters: Humans, English, 2019/01/01–2024/09/15

Supplementary Table S2: Grade Quality assessment for included studies of the meta-analysis

				Certainty Assessment						
Outcomes	n	Study design	RoB	Inconsistency	Indirectness	Imprecision	Other considerations	No. of patients	Effects	Certainty
Weight maintenance	7	RCT	No serious	No serious	No serious	No serious	none	622	SMD = 0.171 (95% CI: 0.008 to 0.335), p=0.040	Moderate
FFMI	3	RCT	serious	No serious	No serious	Serious (small sample size, CI crosses 0)	none	217	SMD = 0.230 (95% CI: –0.049 to 0.509), p=0.107	Low
Albumin	5	RCT	Serious	Serious	Serious	No serious	none	512	SMD = 0.539 (95% CI: 0.150 to 0.927), p=0.007	Very Low
Hemoglobin	3	RCT	Serious	Very Serious (very high heterogeneity)	Serious	Serious (small sample, wide CI)	none	326	SMD = 0.755 (95% CI: –0.411 to 1.921), p=0.205	Very Low
Pre-albumin	3	RCT	Serious	Serious (very high heterogeneity)	No Serious	Serious (small sample size, wide CI, effect driven by single study)	none	314	SMD = 1.092 (95% CI −0.452 to 2.636), p=0.166	Very low
PG-SGA	4	RCT	Serious	Serious	No serious	Serious	none	382	SMD = –0.518 (95% CI: –0.931 to –0.106), p=0.014	Very Low
OM	4	RCT	Serious (narrative synthesis)	Serious (inconsistent findings)	Serious (different grading systems)	Serious (small sample size)	none	234	Not pooled (Narrative synthesis)	Very Low
QoL	3	RCT	Serious (methodological limitations)	Serious (inconsistent outcomes)	Serious (different measurement tools)	Serious (small sample size)	none	202	Not pooled (Narrative synthesis)	Very low

Abbreviations. FFMI: Fat Free Mass Index, PG-SGA: Patient generated subjective global assessment, OM: Oral mucositis. QoL: Quality of life. [Downgraded for RoB due to allocation concealment, downgraded for inconsistency due to high heterogeneity, downgraded for indirectness because albumin/Hb are non-specific nutrition markers].

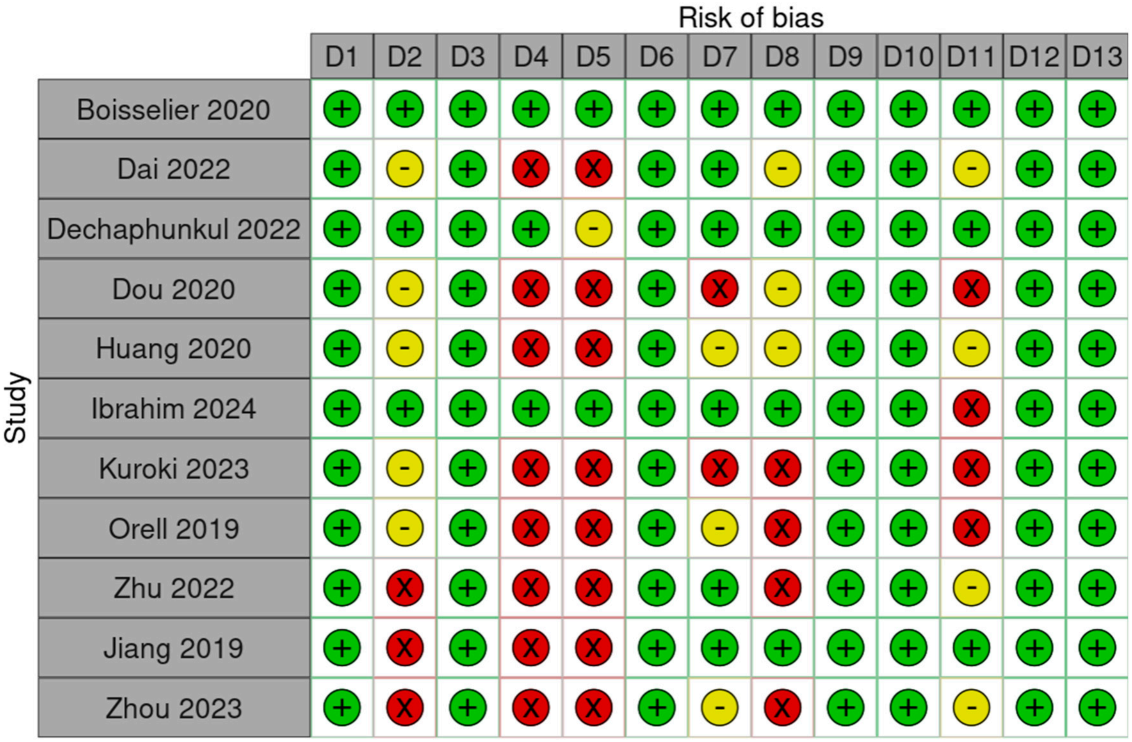

D1	True randomization
D2	Treatment allocation concealment
D3	Groups similar at baseline"
D4	Participant blinding
D5	Therapist blinding
D6	Identical group treatments
D7	Follow up completion, description
D8	Assessors blinding
D9	Outcomes measured in same way
D10	Reliability of outcome measures
D11	Participant analysis in original group
D12	Appropriate statistical analysis
D13	Appropriate trial design

Judgement
X High
- Unclear
+ Low

Supplementary Figure S1: Risk of bias summary for the included studies, based on JBI Checklist for RCTs, visualized using robvis traffic-light plot, with custom mapping for each checklist item.

a. weight

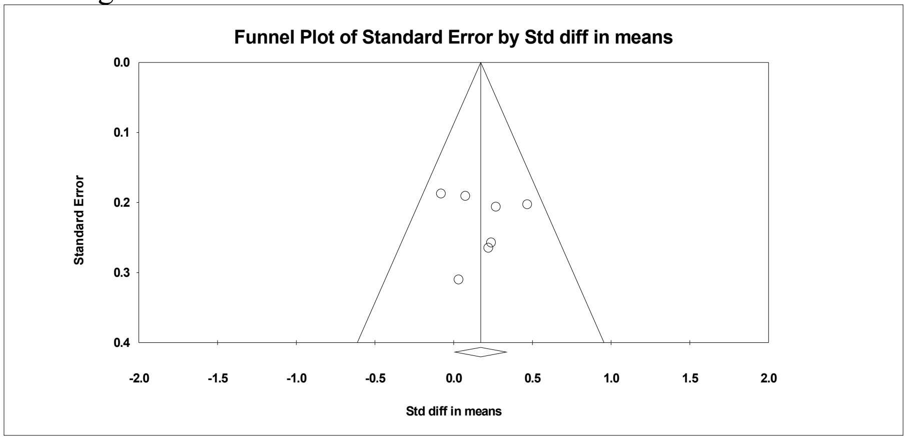

b. Fat Free Mass Index

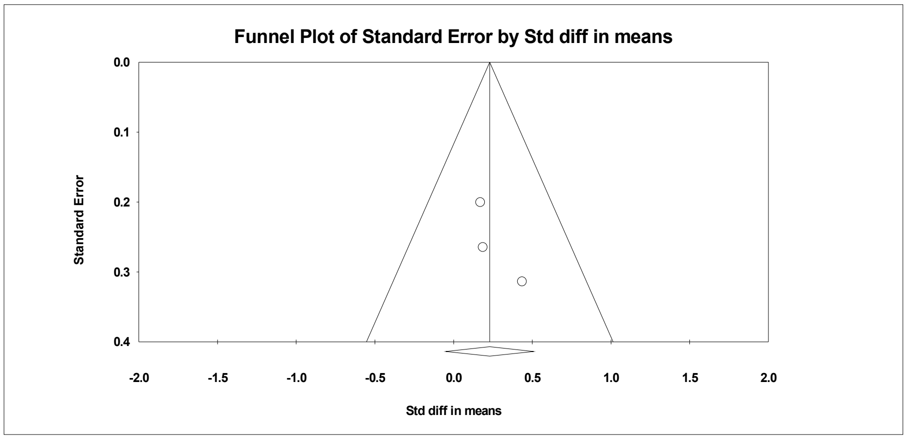

c. PG-SGA

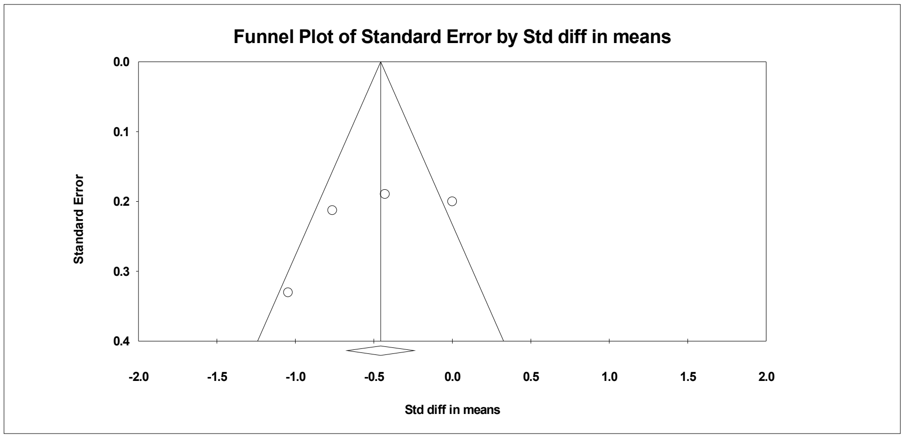

Supplementary Figure S2-1: Funnel Plots of Included Studies (a. weight; b. FFMS; c. PG-SGA)

d. hemoglobin

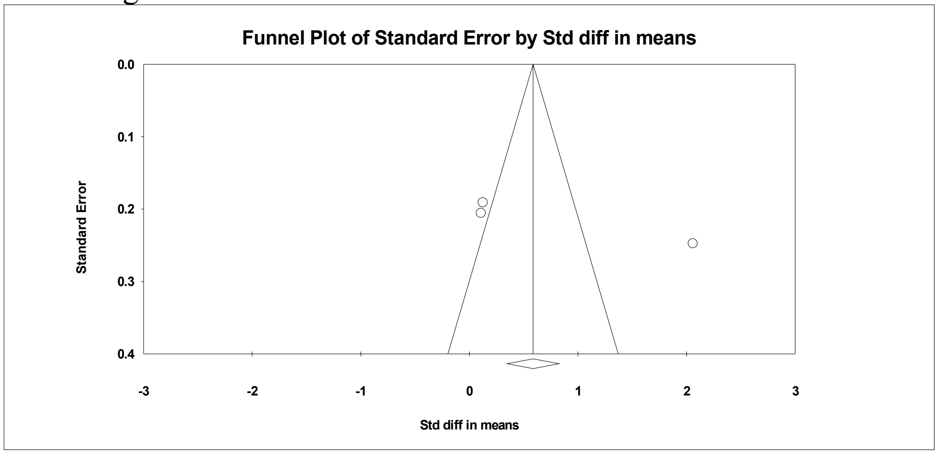

e. albumin

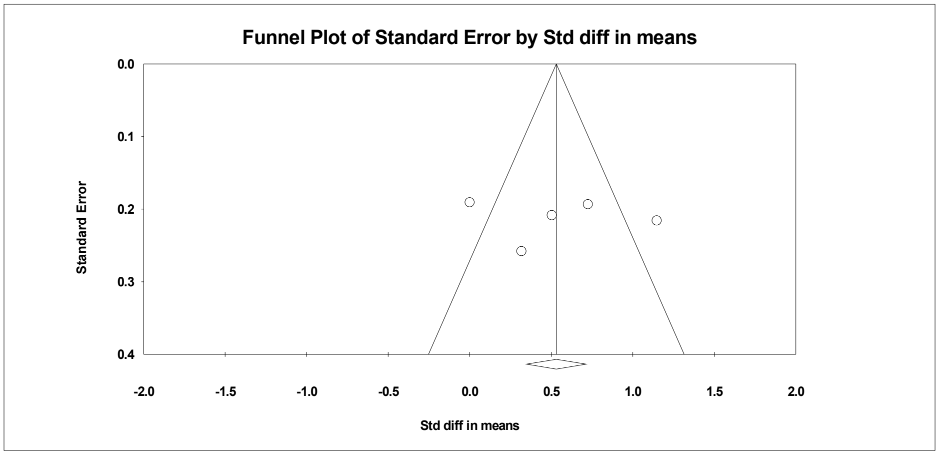

f. pre-albumin

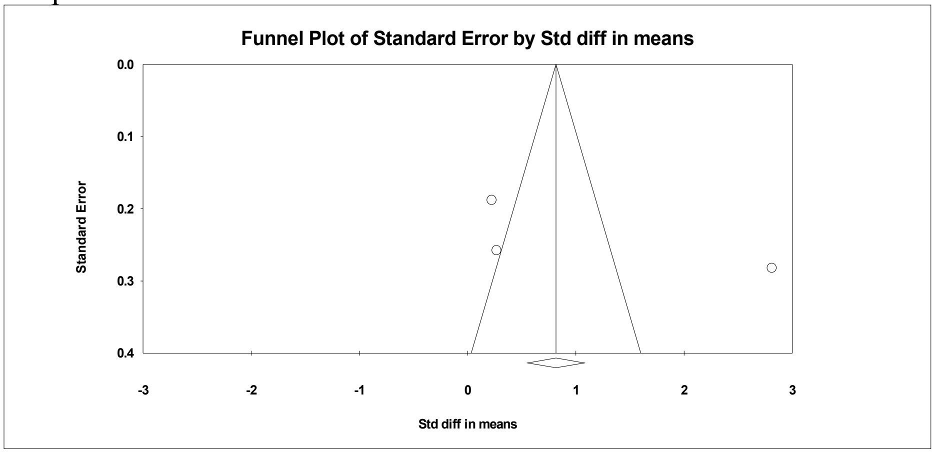

Supplementary Figure S2-2: Funnel Plots of Included Studies (d. hemoglobin; e. albumin; f. pre-albumin)

PRISMA CHECKLIST

Section and Topic	Item #	Checklist item	Location where item is reported
**TITLE**			
Title	1	Identify the report as a systematic review.	p.1
**ABSTRACT**			
Abstract	2	See the PRISMA 2020 for Abstracts checklist.	p.1
**INTRODUCTION**			
Rationale	3	Describe the rationale for the review in the context of existing knowledge.	p.2
Objectives	4	Provide an explicit statement of the objective(s) or question(s) the review addresses.	p.2
**METHODS**			
Eligibility criteria	5	Specify the inclusion and exclusion criteria for the review and how studies were grouped for the syntheses.	p.3
Information sources	6	Specify all databases, registers, websites, organisations, reference lists and other sources searched or consulted to identify studies. Specify the date when each source was last searched or consulted.	p.3, supplementary table 1
Search strategy	7	Present the full search strategies for all databases, registers and websites, including any filters and limits used.	p.3, supplementary table 1
Selection process	8	Specify the methods used to decide whether a study met the inclusion criteria of the review, including how many reviewers screened each record and each report retrieved, whether they worked independently, and if applicable, details of automation tools used in the process.	p.3,4 figure 1
Data collection process	9	Specify the methods used to collect data from reports, including how many reviewers collected data from each report, whether they worked independently, any processes for obtaining or confirming data from study investigators, and if applicable, details of automation tools used in the process.	p.3,4
Data items	10a	List and define all outcomes for which data were sought. Specify whether all results that were compatible with each outcome domain in each study were sought (e.g. for all measures, time points, analyses), and if not, the methods used to decide which results to collect.	p.3,4
	10b	List and define all other variables for which data were sought (e.g. participant and intervention characteristics, funding sources). Describe any assumptions made about any missing or unclear information.	Table 1
Study risk of bias assessment	11	Specify the methods used to assess risk of bias in the included studies, including details of the tool(s) used, how many reviewers assessed each study and whether they worked independently, and if applicable, details of automation tools used in the process.	p.4
Effect measures	12	Specify for each outcome the effect measure(s) (e.g. risk ratio, mean difference) used in the synthesis or presentation of results.	p.4, Figure 2,3,4
Synthesis methods	13a	Describe the processes used to decide which studies were eligible for each synthesis (e.g. tabulating the study intervention characteristics and comparing against the planned groups for each synthesis (item #5)).	p.3,4,Table 1
	13b	Describe any methods required to prepare the data for presentation or synthesis, such as handling of missing summary statistics, or data	N/A

Section and Topic	Item #	Checklist item	Location where item is reported
		conversions.	
	13c	Describe any methods used to tabulate or visually display results of individual studies and syntheses.	p.4
	13d	Describe any methods used to synthesize results and provide a rationale for the choice(s). If meta-analysis was performed, describe the model(s), method(s) to identify the presence and extent of statistical heterogeneity, and software package(s) used.	p.4
	13e	Describe any methods used to explore possible causes of heterogeneity among study results (e.g. subgroup analysis, meta-regression).	p.4
	13f	Describe any sensitivity analyses conducted to assess robustness of the synthesized results.	p.4
Reporting bias assessment	14	Describe any methods used to assess risk of bias due to missing results in a synthesis (arising from reporting biases).	N/A
Certainty assessment	15	Describe any methods used to assess certainty (or confidence) in the body of evidence for an outcome.	p.4
**RESULTS**			
Study selection	16a	Describe the results of the search and selection process, from the number of records identified in the search to the number of studies included in the review, ideally using a flow diagram.	p. 4,5, Figure 1
	16b	Cite studies that might appear to meet the inclusion criteria, but which were excluded, and explain why they were excluded.	Figure 1
Study characteristics	17	Cite each included study and present its characteristics.	Table 1
Risk of bias in studies	18	Present assessments of risk of bias for each included study.	Supplementary figure 1
Results of individual studies	19	For all outcomes, present, for each study: (a) summary statistics for each group (where appropriate) and (b) an effect estimate and its precision (e.g. confidence/credible interval), ideally using structured tables or plots.	Figure 2-4
Results of syntheses	20a	For each synthesis, briefly summarise the characteristics and risk of bias among contributing studies.	p.14 supplementary figure 1
	20b	Present results of all statistical syntheses conducted. If meta-analysis was done, present for each the summary estimate and its precision (e.g. confidence/credible interval) and measures of statistical heterogeneity. If comparing groups, describe the direction of the effect.	p.10,11,12,13 Figure 2, 3, 4
	20c	Present results of all investigations of possible causes of heterogeneity among study results.	p. 15
	20d	Present results of all sensitivity analyses conducted to assess the robustness of the synthesized results.	p. 15
Reporting biases	21	Present assessments of risk of bias due to missing results (arising from reporting biases) for each synthesis assessed.	N/A
Certainty of evidence	22	Present assessments of certainty (or confidence) in the body of evidence for each outcome assessed.	p.14, 15 Table 2
**DISCUSSION**			

Section and Topic	Item #	Checklist item	Location where item is reported
Discussion	23a	Provide a general interpretation of the results in the context of other evidence.	p.15,16,17
	23b	Discuss any limitations of the evidence included in the review.	p.17
	23c	Discuss any limitations of the review processes used.	p.17
	23d	Discuss implications of the results for practice, policy, and future research.	p.16,17
**OTHER INFORMATION**			
Registration and protocol	24a	Provide registration information for the review, including register name and registration number, or state that the review was not registered.	p. 2,3
	24b	Indicate where the review protocol can be accessed, or state that a protocol was not prepared.	p. 2,3
	24c	Describe and explain any amendments to information provided at registration or in the protocol.	N/A
Support	25	Describe sources of financial or non-financial support for the review, and the role of the funders or sponsors in the review.	p.17
Competing interests	26	Declare any competing interests of review authors.	p.18
Availability of data, code and other materials	27	Report which of the following are publicly available and where they can be found: template data collection forms; data extracted from included studies; data used for all analyses; analytic code; any other materials used in the review.	p. 5, Table 1
